# Inability of Some Commercial Assays to Measure Suppression of Glucagon Secretion

**DOI:** 10.1155/2016/8352957

**Published:** 2015-12-29

**Authors:** Nicolai J. Wewer Albrechtsen, Simon Veedfald, Astrid Plamboeck, Carolyn F. Deacon, Bolette Hartmann, Filip K. Knop, Tina Vilsboll, Jens J. Holst

**Affiliations:** ^1^Department of Biomedical Sciences, Faculty of Health and Medical Sciences, University of Copenhagen, 2200 Copenhagen, Denmark; ^2^Novo Nordisk Foundation Center for Basic Metabolic Research, Faculty of Health and Medical Sciences, University of Copenhagen, 2200 Copenhagen, Denmark; ^3^Center for Diabetes Research, Gentofte Hospital, University of Copenhagen, 2900 Hellerup, Denmark

## Abstract

Glucagon levels are increasingly being included as endpoints in clinical study design and more than 400 current diabetes-related clinical trials have glucagon as an outcome measure. The reliability of immune-based technologies used to measure endogenous glucagon concentrations is, therefore, important. We studied the ability of immunoassays based on four different technologies to detect changes in levels of glucagon under conditions where glucagon levels are strongly suppressed. To our surprise, the most advanced technological methods, employing electrochemiluminescence or homogeneous time resolved fluorescence (HTRF) detection, were not capable of detecting the suppression induced by a glucose clamp (6 mmol/L) with or without atropine in five healthy male participants, whereas a radioimmunoassay and a spectrophotometry-based ELISA were. In summary, measurement of glucagon is challenging even when state-of-the-art immune-based technologies are used. Clinical researchers using glucagon as outcome measures may need to reconsider the validity of their chosen glucagon assay. The current study demonstrates that the most advanced approach is not necessarily the best when measuring a low-abundant peptide such as glucagon in humans.

## 1. Introduction

Glucagon, a 29-amino-acid peptide secreted from the pancreatic alpha cells in response to hypoglycemia [1], is derived from the proglucagon molecule, which is also expressed in the intestine and brain [2]. Glucagon has stimulatory effect on hepatic glucose production, and dysregulation of its secretion may contribute to the development of diabetes [3–6]. Glucagon measurements are, therefore, often an important study outcome; according to clinicaltrials.gov, it is included as an endpoint in more than 400 clinical studies. However, measurement of glucagon is a delicate matter and the validity of the data relies on sufficient specificity and sensitivity of the assay. Differential tissue-specific processing of proglucagon results in molecular heterogeneity, meaning that assay specificity with respect to the different molecular forms is important. Thus, in addition to glucagon itself, proglucagon gives rise to several peptides containing the glucagon sequence, including oxyntomodulin, glicentin, and proglucagon 1–61, as well as molecules with some sequence homology to glucagon, including glucagon-like peptide-1 (GLP-1) and glucagon-like peptide-2 (GLP-2) and major proglucagon fragment [7]. Furthermore, each of these molecular forms may occur in extended or truncated forms, which may or may not be biologically active [2]. The immediate specificity problem is therefore of considerable magnitude. Sensitivity is equally important, since glucagon occurs in low picomolar concentrations in the circulation. Its concentration rises in response to hypoglycemia and falls in response to rising glucose (e.g., after carbohydrate meals), with the rate of as well as the absolute magnitude of the decrease being of considerable importance for the resulting glucose tolerance. The ability of assays to register these decreases from already low levels is, therefore, critical [8].

In the current study, we investigated assays based on four widely applied immune-based technologies: a radioimmunoassay (RIA), a spectrophotometric enzyme-linked immunoassay (ELISA), and ELISAs based on electrochemiluminescence (ECL), and homogeneous time-resolved fluorescence (HTRF) detection. We hypothesized that the assay type might influence measured glucagon concentrations. To address this, we analyzed glucagon levels during a glucose clamp with or without atropine (atropine blocks cholinergic signaling through the muscarinic receptors and leads to further suppression of glucagon secretion) in five healthy male participants using these four different approaches; previous measurements indicated that the clamp + atropine protocol resulted in pronounced suppression of glucagon levels [9].

## 2. Methods

### 2.1. Participants, Procedures, and Samples

Samples were derived from a previously published study by Plamboeck et al. [9]. The study was conducted in accordance with the Helsinki Declaration II and was approved by the Scientific-Ethical Committee of the Capital Region of Denmark (registration number: H-2-2011-062) and by the Danish Data Protection Agency (journal number: 2011-41-6381) and registered at clinicaltrials.gov (ID: NCT01534442). Oral and written informed consent was obtained from all participants. Glucose clamps (6 mmol/L) were performed in five healthy male participants (age: 25 ± 1 years, body mass index: 24 ± 0.5 kg/m^2^, and HbA_1c_: 5.1 ± 1%) with or without blocking efferent muscarinic activity by infusion of atropine (1 mg bolus + an 80 ng/kg/min infusion). Samples were collected and stored using optimal conditions for glucagon analysis as described previously [8].

### 2.2. Measurement of Glucagon

We used four immune-based assays for measurement of glucagon: (A) an in-house C-terminal RIA (codename 4305) [6, 8, 10]; (B) Mercodia sandwich ELISA (spectrophotometry) (cat# 10-1271-01, Uppsala, Sweden); (C) sandwich ELISA from MSD (chemiluminescence) (cat# K151HCC-1, MD 21201, USA); and (D) sandwich ELISA from Cis-Bio (homogeneous time-resolved fluorescence) (cat# 62GLCPEK, Codolet, France). Assays were carried out as per protocol according to the manufacturers' instructions. Samples were kept cold (ice-bath) at all times, and all samples were measured simultaneously in a single run to eliminate interassay variance.

### 2.3. Statistics

To analyze changes in glucagon levels over time, a one-way ANOVA for repeated measurements followed by a Bonferroni post hoc analysis was performed for each of the four assays. To compare the ability of the assays to detect changes in glucagon levels, we created a generalized regression model (ANCOVA) with glucagon as dependent variable and time (minutes) and method (assay) as independent variables. Net area under the curve (delta changes from time zero to 160 minutes relative to the individual baselines) (nAUC) was calculated using the trapezoidal rule and differences were tested using a two-sided test. A power calculation was made based on the following assumptions: normality of data distribution, homoscedasticity, one-sample *t*-test, quantification limits and coefficient of variations* provided by the manufacturers*, an alpha value of 0.05, and a sample size of five. The calculation showed that the power to detect significant changes (of 5%) in glucagon levels ranged from 79% to 84% across the four assays. *P* < 0.05 was considered significant. Calculations were made using GraphPad Prism version 6.04 for Windows, GraphPad Software, La Jolla, California, USA, http://www.graphpad.com/, and STAT14, Boston, MA, USA. For illustrations we used the Adobe CS6 software suite (California, USA).

## 3. Results

**Figure 1:**
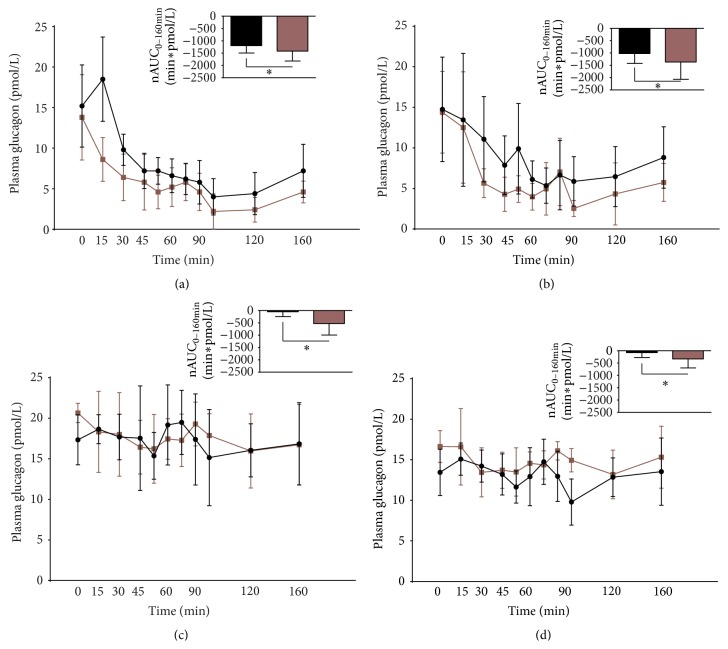
Plasma glucagon levels of five healthy participants during a 6 mmol/L glucose clamp with simultaneous infusion of either saline (black) or atropine (red). (a) depicts assay A, a radioimmunoassay; (b) depicts assay B, a spectrophotometrically based ELISA; (c) depicts assay C, a chemiluminescence based ELISA; and (d) depicts assay D, a homogeneous time-resolved fluorescence based ELISA. Net area under the curve (nAUC) is depicted separately at upper right quadrant on (a), (b), (c), and (d). *∗* represents a significant two-sided *t*-test comparing saline nAUC to atropine nAUC. Data illustrated as mean ± standard deviation.

The recoveries of synthetic glucagon in pooled human plasma (*N* = 4) were 95 ± 11% (assay A), 104 ± 5% (assay B), 75 ± 15% (assay C), and 67 ± 21% (assay D). Glucagon levels dropped significantly compared to baseline (time = 0 min) in both saline and atropine treated groups (*P* < 0.01) when samples were measured using assay A (Figure 1(a)) and assay B (Figure 1(b)) but not with assay C (Figure 1(c), *P* = 0.31) and assay D (Figure 1(d), *P* = 0.24). Assay A was significantly different (*P* < 0.05) from assays C and D but not assay B (*P* = 0.43). Assay B was significantly different from assays C and D (*P* < 0.05) whereas there were no differences between assays C and D (*P* = 0.27). nAUCs during infusion of saline and atropine, respectively, for assay A and assay B were significantly different (*P* < 0.001), indicating further suppression of glucagon secretion with atropine addition. For assays C and D, nAUCs were significantly different between atropine (*P* < 0.01) and saline, indicating that atropine weakly suppressed glucagon levels compared to the clamp alone, where nAUCs did not show significance compared to baseline (zero) by one-side *t*-test (*P* = 0.11 and *P* = 0.17).

## 4. Discussion

Immune-based detection methods utilize the extreme binding energy of antibodies which may have equilibrium constants reaching values of 10^12^ L/mol, providing these methods with a potential to measure very low concentrations. However, the use of antibodies relies on their specificity and the antigen-antibody reaction may also may be sensitive to the so-called matrix effects, that is, interference from components in plasma including a variety of high-abundant plasma molecules or proteins (e.g., albumin and immunoglobulins), leading to unspecific interference in antibody-antigen interaction [11].

Assay D uses the homogeneous time-resolved fluorescence technology which combines fluorescence resonance energy transfer technology (FRET) with time-resolved measurement (TR) [12]. HRTF is mainly used in (*in vitro*) primary and secondary screening phases of drug development. However, its usefulness in highly sensitive immunoassays required for detection of 1 pmol/L differences in glucagon levels may be questioned. In contrast, assay C applies an electrochemiluminescence approach: when excited by electrical stimulation, labeled molecules emit light, which then is detected by cameras. The most generic ELISA used in our study is assay B, involving spectrophotometry detection; a chromogenic substrate is added to sample wells, which is then cleaved by an enzyme, for example, horseradish peroxidase, coupled to the detection antibody. Assay A is a radioimmunoassay utilizing competition between radioactively labeled antigen and unlabeled antigen (peptide standard or sample with unknown concentration) for binding to a limited number of specific antibody binding sites. Although it is the most simple, with regard to technology, the data clearly shows that assays A and B perform significantly better than assays C and D. However, a requirement for such a performance is the application of antibodies with sufficient binding energy (and specificity), which is often the crucial step in assay development.

In this study, we highlight the crucial importance of choosing the “right” immune-based method when analyzing endogenous glucagon. We have exemplified the challenge by demonstrating that changes in measured glucagon levels depend on the assay used (Figure 1); where assays A and B clearly register the attenuation of glucagon levels during a glucose clamp, assays C and D did not. The basal levels measured were comparable, around 15 pmol/L (although assay C showed slightly higher levels, around 20 pmol/L). This may reflect a specificity problem in assay C; for example, this assay could be cross-reacting with glucagon-like molecules (oxyntomodulin, glicentin, or glucagon-like peptide-1 [7, 11]) although not stated by the manufacturers. Otherwise, the difference between the assays, although not formally tested here, most likely reflects differences in sensitivity and therefore ability to detect dynamic changes in the very low picomolar range. In a previous study [6], a sensitivity analysis was carried out for assays A and B (where assay A was more sensitive), and, in another study of other commercially available assays [7], sensitivity was clearly insufficient to allow analysis in this concentration range. In addition, we recently demonstrated that measured glucagon levels in subjects with renal dysfunction may erroneously appear elevated, probably due to cross-reactions with N-terminal elongated inactive isoforms of the glucagon molecule (1–61) when analyzed with conventional single antibody C-terminal radioimmunoassays [6]. Importantly for interpretation of clinical studies, the potential instability of glucagon during inappropriate sample preparation and storage, such as multiple freeze-thaw cycles or storing plasma samples at room temperature for more than 1 hour, should also be considered [8].

Novel mass-spectrometry based detection (e.g., selected reaction monitoring (SRM)) of low-abundant peptides as glucagon [13] may in the future facilitate validation of immune-based detection methods. Unfortunately, current mass-spectrometry based methods still depend on 2D-gel extraction techniques or bead-coupled antibodies [14] both of which have questionable recoveries and specificity. However, in the future mass-spectrometry based detection methods may involve label-free (be it chemical or antibody based) purification steps as recently demonstrated [15] and may therefore provide accurate validation.

In conclusion, levels of glucagon are increasingly being used as outcome measures in clinical trials and the reliability of the glucagon assays employed is therefore critical for appropriate interpretation of the data.
